# Obstetrics and Gynecology and Reparations: The Debt We Owe (and Continue to Accumulate)

**DOI:** 10.1089/heq.2021.0015

**Published:** 2021-05-24

**Authors:** Megan L. Swanson, Sara Whetstone, Tushani Illangasekare, Amy (Meg) Autry

**Affiliations:** Division of Gynecologic Oncology, Department of Obstetrics, Gynecology and Reproductive Sciences, University of California San Francisco, San Francisco, California, USA.

**Keywords:** health disparities, women's health, reparations

## Abstract

Obstetrics and gynecology (OBGYN) is rife with exploitation and oppression of Black individuals and disparate health outcomes. We posit that racial disparities in OBGYN are fueled by racism and the racial wealth gap stemming from slavery, legal segregation, and institutionalized discrimination against Black Americans. We believe reparations are not only morally requisite, but would also improve health outcomes for our patients. Supporting legislation to explore and remedy the harms of slavery and its legacy is critical to address systemic racism that results in disparate health outcomes.

Marion Sims performed experimental surgery on enslaved Black women without anesthesia. Researchers utilized the bodily tissue of Henrietta Lacks without permission or compensation. Doctors performed a “Mississippi appendectomy” on Fannie Lou Hamer, sterilizing her without her knowledge or consent. These are just three well-known examples of how the bodies of Black women were exploited in the practice of obstetrics and gynecology (OBGYN). The field of OBGYN has a history of systematic abuse and oppression of Black, Indigenous, and People of Color (BIPOC) patients and is a field of medicine that is rooted in White supremacy and racism. Examples of mistreatment of BIPOC individuals by our specialty are not consigned to history. The assault on Black and Brown bodies continues with forced sterilizations of 20,000 women in state institutions in California,^1–3^ and, most recently, with high rates of hysterectomy procedures performed on immigrants held in Immigration and Customs Enforcement custody.^4,5^

OBGYN has some of the most enduring inequities in health outcomes. Black women in the United States are more than three times more likely to experience a pregnancy-related death than White women even when controlling for education and income.^6^ Black women have higher rates of preterm birth and small for gestational age neonates,^7^ and Black infants have a mortality rate two times that of White infants.^8,9^ Black women suffer disproportionately more morbidity and mortality rates from cervical, endometrial, and ovarian cancers with worse overall survival in all major gynecological malignancies compared with their White counterparts.^10^ Black women experience a higher incidence of infertility,^11^ and lower odds of achieving pregnancy, and live birth through assisted reproductive technology.^12^ These and countless other OBGYN disparities can be traced to racism perpetrated by the field itself and by society at large.^13^

For decades, medicine propagated the premise that there were innate intrinsic differences among Blacks that accounted for these shockingly disparate outcomes. It is now clear that racism and oppression, not race, drive health inequities.^14^ Because of the impact of structural violence on health, it is impossible to eliminate disparities in health outcomes by race without addressing the structural problem of systemic societal racism. If we are serious about equity and justice in health, we need to look beyond individual patient care and beyond health care in general—to societal transformation.

We posit that racial inequities in wealth are an important factor driving the racial disparities in OBGYN outcomes that persist even when controlling for measurable confounders such as age, education, and income. For centuries, White Americans have benefited from national policies, including the New Deal Federal Housing Administration home loans notoriously distributed based on racist redlining maps, that have contributed to the creation of an extreme wealth gap wherein the average Black family holds one penny of wealth for every dollar of the average White family.^15^ If we agree that it is racism, not race, contributing to these wealth and health disparities, then we are compelled to consider a process of reconciliation and redress, including material reparations. And we, as physicians, need to use our positions of power as advocates of justice.

Although nothing can rectify the atrocities of slavery and the legal segregation and discrimination against Black Americans upheld by our federal government for more than a century after the abolition of slavery, there is precedent for compensating individuals and funding development for communities wronged. Although the federal government has refused to consider reparations for enslaved persons (a 30-year-old bill calling for congressional study of slavery and its effects as well as recommendations for remediation has never made it to the House floor for a vote), it has passed legislation and issued payments to Native Americans and Native Hawaiians for unjust seizure of lands, to Japanese people interned during World War II, and to victims of the Tuskegee experiments and the Rosewood massacre. More recently, in 2020, the city council of Asheville, North Carolina, passed a resolution for community reparations to establish a process addressing the creation of generational wealth and boosting economic mobility and opportunity in the Black community. Also in 2020, the state legislature of California passed a bill to formally study reparations for Black Californians.

Although there is no direct evidence demonstrating exactly how reparations would affect health disparities, data show that monetary benefits improve outcomes in OBGYN, especially for BIPOC patients. The earned income tax credit (EITC), the largest poverty alleviation program in the United States, has been consistently associated with improved maternal and child health outcomes.^16–19^ Although EITC supplements improve birth outcomes across all racial subgroups, the largest beneficial effect has been observed for Black women.^20^ Modeling studies have demonstrated that EITC supplements are cost-effective in terms of increasing health-related quality of life and longevity.^21^ Data from Canada show that an unconditional prenatal income supplement for low-income women resulted in significantly improved birth outcomes and population-level decreases in birth–outcome inequities.^22^ Acknowledging these data and recognizing the relationship of the wealth gap to higher maternal and infant mortality rates among Black and Pacific Islander families in San Francisco, Mayor London Breed recently announced a pilot program to provide basic income during pregnancy and postpartum.

Data associating unconditional income supplementation to improved OBGYN outcomes cannot explain exactly why BIPOC patients are experiencing particular benefit, though there are theories. Drs. Bassett and Galea recently argued that addressing the Black–White wealth gap, one of the underlying causes of racial health disparities, through reparations for slavery could help reduce health disparities and they posit three mechanistic pathways: expanding resources available to Black Americans, reducing stress experienced by Black Americans, and/or the intergenerational effect of Black reparations translating into wealth for this and subsequent generations.^23^

In a recently released collective statement by 19 OBGYN professional organizations, including the American College of Obstetricians and Gynecologists, the leadership of our specialty acknowledged “the injustices inextricably linked to the field of obstetrics and gynecology [and the need to] recognize all the contributions made both willingly and unwillingly by oppressed and marginalized persons.” We must confront the history of our specialty and our role in perpetuating harm to BIPOC communities. We must acknowledge that BIPOC providers, especially midwives, were systematically excluded through legislative advocacy of White male gynecologists and the reverberations of that exclusion continue to impact reproductive health outcomes today. We must recognize that the reproductive justice movement, asserting the human right to sexuality, gender, work, and reproduction, was a response to a system that denied women of color the right to bodily autonomy in reproductive decision making. We must admit that today, obstetric racism, the intersection of obstetric violence and medical racism, continues to jeopardize the lives of Black birthing individuals.

As health care providers, we have a moral imperative to advocate for just policies to promote equity for our patients. We must actively dismantle racism, the root of inequity, and atone for the destruction of communities, the loss of lives, and the denial of human rights that inevitably accompany racism. The Commission to Study and Develop Reparation Proposals for African-Americans Act (H.R.40/S.B.1083) is an example of proposed congressional legislation that would establish a commission to “examine slavery and discrimination in the colonies and the United States from 1619 to the present and recommend appropriate remedies.” This bill was just reintroduced in the House of Representatives and proponents are hopeful for support from the Biden administration, noting the administration's public commitment to tackle structural oppression.

The aforementioned OBGYN collaborative statement calls for policy and advocacy on the part of physicians, including advocating for policies addressing systemic and institutional inequities that lead to poor health outcomes. Supporting H.R.40/S.B.1083 is an example of the advocacy called for in this statement. The specialty of OBGYN, which has perpetuated individual and systemic harm since its inception, owes a debt too large to estimate to our BIPOC patients, communities, and colleagues. As professionals who espouse science and evidence, we must reckon with the effects of slavery and discrimination and investigate the process of reconciliation and reparations. It is our responsibility to advocate for our patients and support legislation advancing equity, centering justice, and addressing the root of racism, a public health crisis.

## Abbreviations Used

BIPOC: Black, Indigenous, and People of Color
EITC: earned income tax credit
OBGYN: obstetrics and gynecology
